# Using Brain Tumor MRI Structured Reporting to Quantify the Impact of Imaging on Brain Tumor Boards

**DOI:** 10.3390/tomography9020070

**Published:** 2023-04-18

**Authors:** Syed A. Abidi, Michael J. Hoch, Ranliang Hu, Gelareh Sadigh, Alfredo Voloschin, Jeffrey J. Olson, Hui-Kuo G. Shu, Stewart G. Neill, Brent D. Weinberg

**Affiliations:** 1 Mallinckrodt Institute of Radiology, Washington University, St. Louis, MO 63110, USA; 2 Department of Radiology, University of Pennsylvania, Philadelphia, PA 19104, USA; 3 Department of Radiology and Imaging Sciences, Emory University, Atlanta, GA 30322, USA; 4 Department of Radiology, University of California-Irvine, Irvine, CA 92868, USA; 5 Department of Neuro-Oncology, Orlando Health Cancer Institute, Orlando, FL 32806, USA; 6 Department of Neurosurgery, Emory University, Atlanta, GA 30322, USA; 7 Department of Radiation Oncology, Emory University, Atlanta, GA 30322, USA; 8 Department of Pathology, Emory University, Atlanta, GA 30322, USA

**Keywords:** structured reporting, brain tumor, glioblastoma, tumor board, multidisciplinary

## Abstract

Multidisciplinary tumor boards (TB) are an essential part of brain tumor care, but quantifying the impact of imaging on patient management is challenging due to treatment complexity and a lack of quantitative outcome measures. This work uses a structured reporting system for classifying brain tumor MRIs, the brain tumor reporting and data system (BT-RADS), in a TB setting to prospectively assess the impact of imaging review on patient management. Published criteria were used to prospectively assign three separate BT-RADS scores (an initial radiology report, secondary TB presenter review, and TB consensus) to brain MRIs reviewed at an adult brain TB. Clinical recommendations at TB were noted and management changes within 90 days after TB were determined by chart review. In total, 212 MRIs in 130 patients (median age = 57 years) were reviewed. Agreement was 82.2% between report and presenter, 79.0% between report and consensus, and 90.1% between presenter and consensus. Rates of management change increased with increasing BT-RADS scores (0—3.1%, 1a—0%, 1b—66.7%, 2—8.3%, 3a—38.5%, 3b—55.9, 3c—92.0%, and 4—95.6%). Of 184 (86.8%) cases with clinical follow-up within 90 days after the tumor board, 155 (84.2%) of the recommendations were implemented. Structured scoring of MRIs provides a quantitative way to assess rates of agreement interpretation alongside how often management changes are recommended and implemented in a TB setting.

## 1. Introduction

Multidisciplinary tumor boards (TBs) are an essential component of NIH National Cancer Institute-designated cancer centers. They provide a team approach to discuss diagnoses and recommend treatment options for patients and are typically subspecialized depending on the type of cancer [1]. The value of TBs has previously been reported in head and neck [2], gastrointestinal [3], and lung cancers [4]. The goals of TBs include determining treatment plans, making management changes, and re-evaluating diagnoses [5,6,7]. TBs are associated with high rates of adherence to National Comprehensive Cancer Network guidelines [3] and implementation of clinical recommendations [8,9,10].

Imaging review is a crucial part of TBs, which can change the initial diagnosis, guide surgical management, and determine therapeutic plans [11]. Unambiguous interpretation of imaging is key for appropriate decision-making, and the stakes are higher for cancers with historically poor prognosis, such as primary brain tumors [12], when there are limited opportunities to change therapy or enroll in clinical trials. Each year, over 80,000 patients in the United States and nearly 350,000 patients worldwide are diagnosed with new brain tumors [13,14]. Glioblastomas are the most common primary brain tumors, and have a dismal prognosis with overall survival less than 2 years even with maximal treatment [15]. Brain tumor patients such as these require frequent management decisions that are often made based on interpreting imaging in the context of TBs. The free-text format of traditional radiology reports can complicate decision-making if they do not provide organized and unambiguous information that can be easily translated into clinical decisions [16,17]. Structured reporting and report scoring can decrease the ambiguity of radiology reports to better guide management [18], and has become widely accepted for a variety of malignancies, such as breast [19] and head and neck [20] cancers. The Brain Tumor Reporting and Data System (BT-RADS) is a structured report and scoring system for glial brain tumors and offers an analogous approach to interpret MRIs for patients with glial brain tumors using an organized reporting template, a dedicated section for relevant clinical history, and detailed classification and scoring criteria [21]. Implementing a BT-RADS system at one institution resulted in increased perceptions of report consistency, confidence in report findings, and physician communication [22] and quantitative improvements in report ambiguity, length, and number of addenda [23]. These qualities make the use of BT-RADS ideal for use in a TB setting. 

Though there is a growing body of data regarding TBs and their impact on management decisions and survival for specific subtypes of cancers [2,3,4], there are only sparse data regarding brain TBs. Similarly, there are limited quantitative data about how structured reporting of brain tumors can be used to understand clinical decision-making, particularly in the TB setting. The purpose of this work is to use a structured reporting system for classifying brain tumor MRIs (BT-RADS) to prospectively assess the value of imaging review in a TB setting and its impacts on brain tumor patient management.

## 2. Materials and Methods

This was a single center prospective study that included all consecutive patients presented at an adult brain tumor board at a single tertiary care institution between October 2017 and September 2018. Inclusion criteria were a diagnosis of a primary parenchymal brain tumor presented for review of brain MRI. Exclusion criteria included patient age < 18 years old, diagnosis of metastatic disease or meningioma, or lack of availability of suitable comparison imaging. The study was approved by the Emory University Institutional Review Board and patient consent was waived.

The tumor board included representation from each subspecialty service involving brain tumor care, including neuroradiologists, radiation oncologists, neuro-oncologists, neurosurgeons, and pathologists and met weekly for one hour to review a maximum of 10 cases selected by subspecialized physicians from any of the radiation oncology, neuro-oncology, or neurosurgery clinical services. Each patient was evaluated by the group with review of pertinent patient data, including clinical and medical history, diagnosis, and any past treatment. Relevant current and prior imaging studies were reviewed by the group. If necessary, pathology reports from prior biopsies or surgeries were also reviewed. Pathology was classified according to the WHO 2016 criteria. At the end of the review, a consensus recommendation or range of recommendations in changes of management was made by the group and recorded by the neuroradiologist who participated in the TB (presenting neuroradiologist). TB recommendations were grouped into the following categories: re-biopsy/re-operate, additional testing (e.g., perfusion imaging, spectroscopy), shortened interval follow-up, start of new chemotherapy or radiation therapy, enrollment in a clinical trial, or palliation. Only primary parenchymal glial brain tumors were included for the purpose of this study, although other cancer types (such as meningioma, lymphoma, metastases, etc.) were discussed at the tumor board. 

Each MRI examination reviewed as part of the tumor board was scored at multiple stages using the previously published BT-RADS criteria [21]. First, at the time MRIs were performed as part of clinical care, reports were generated using a BT-RADS-specific dictation template (Powerscribe, Nuance, Burlington, MA, USA) by one of the 16 board-certified neuroradiologists at our institution (report score). At the end of each report, studies were scored on a range from 0 to 4 based on the likelihood of tumor worsening using specific criteria for timing of radiation, administration of medications such as bevacizumab, and worsening of FLAIR and post-contrast T1-weighted imaging (Table 1) [24]. This was recorded as the report score. If the initial interpreting radiologist reported no BT-RADS score in the official report, the presenting neuroradiologist determined which numerical score would have been applied to the report based only on the information present in the initial radiology report. That is, if FLAIR or enhancement was reported to be worse, structured reporting rules were followed based on the initial radiology report regardless of whether the presenting neuroradiologist agreed with the score.

Second, prior to the TB, each case to be presented was reviewed by one of four presenting board-certified neuroradiologists who routinely participate in the TB. This neuroradiologist assigned a second BT-RADS score based on their secondary interpretation and any additional information available at the time of TB review (presenter score). The presenter was not blinded to the radiology report or report score.

Third, after the group TB discussion, a final TB imaging score was assigned based on consensus imaging interpretation by the entire TB group (consensus score). This score was documented by the presenting neuroradiologist at the time of the tumor board. As a result, each MRI was scored on 3 separate occasions: (1) the initial report score, (2) the secondary presenter score, and (3) the tumor board consensus score.

Clinical data including pathology diagnosis, treatment decisions, and outcomes were obtained from retrospective review of the electronic medical record for each patient at least 90 days after TB review. Treatment decisions made after the TB were determined by reviewing the earliest clinical neuro-oncology, radiation oncology, or neurosurgery note occurring after the tumor board date. Results of any additional biopsies performed were recorded and categorized by whether the sample contained >50% tumor cells. Progression-free and overall survival were also recorded where available. Each data entry was subsequently checked by one of the presenting neuroradiologists to confirm accuracy.

Rates of agreement between scores were calculated using total overall agreement rate and linear-weighted kappa (assuming a linear relationship between scores 0 and 4), with ranges shown as the 95% confidence interval. Proportional data were compared using Fisher’s exact test. Statistical significance was determined as *p* < 0.05.

## 3. Results

### 3.1. Patient Demographics

A total of 212 cases meeting the inclusion criteria were presented at an adult brain TB over the one-year period, with 130 unique patients ranging from 23 to 84 years in age. The most common tumor was glioblastoma (*n* = 63, 48.5%), followed by anaplastic astrocytoma (*n* = 16, 12.3%), oligodendroglioma (*n* = 12, 9.2%), low grade astrocytoma (*n* = 11, 8.5%), anaplastic oligodendroglioma (*n* = 10, 7.7%), gliosarcoma (*n* = 4, 3.1%), unbiopsied but presumed gliomas (*n* = 6, 4.6%), and single instances of other tumors as mentioned in Table 2. 

### 3.2. Score Comparison and Concordance

**Figure 1:**
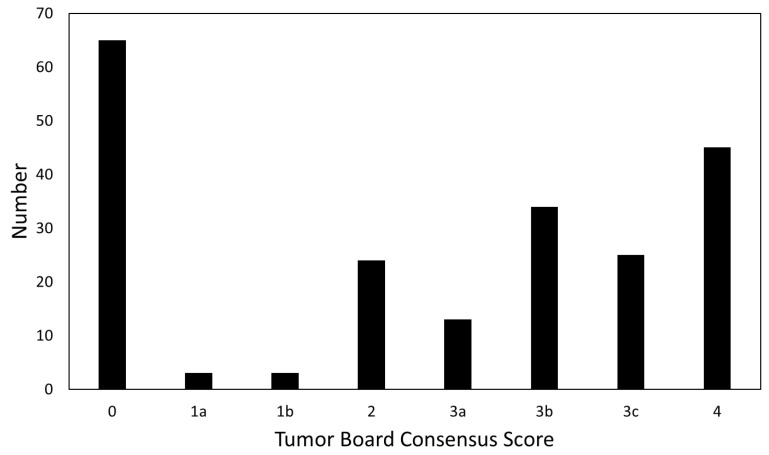
The distribution of tumor board consensus scores for 212 cases presented.

**Figure 2:**
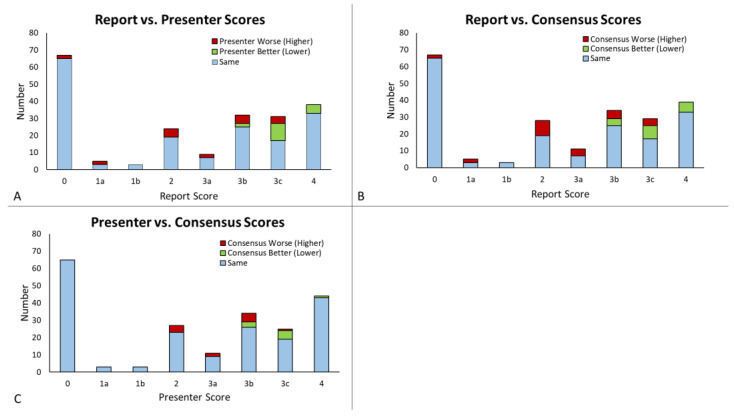
Frequency of agreement between report, presenter, and consensus MRI scores. (**A**) The report scores compared with the presenter scores, with higher presenter scores shown in red and lower presenter scores shown in green. (**B**) The report scores compared with the consensus score, with higher consensus scores shown in red and lower consensus scores shown in green. (**C**) Presenter scores compared with the consensus score, with higher consensus scores shown in red and lower consensus scores shown in green.

The distribution of tumor board consensus scores for all 212 cases is shown in Figure 1. The most common scores were 0, most frequently assigned during first presentation for determination of initial management, followed by 4, high likelihood of tumor progression. In total, 209 cases (98.6%) had the original radiology report available; 134 (63.2%) included a BT-RADS score and 75 (35.3%) did not. Three cases had no original radiology reports available (uploaded cases from another institution), and no report score was given. All 212 cases (100%) were assigned a presenter and tumor board consensus score. Agreement between groups is shown in Figure 2. There was an 82.2% overall agreement among the report and presenter scores, with presenter score being more favorable (lower score) in 8.1% of cases and less favorable (higher score) in 9.7% of cases (Figure 2A). Report and presenter scores had a linear-weighted kappa of 0.88 ± 0.06. Among the report and consensus scores, there was a 79.0% agreement, with the consensus scores being more favorable 8.6% and less favorable 12.4% of the time (Figure 2B). Linear-weighted kappa between the report and consensus scores was 0.86 ± 0.06. When comparing the presenter to the consensus scores, there was 90.1% agreement among all 212 scores, with the consensus score being more favorable in 4.2% and less favorable in 5.7% of cases (Figure 2C). The presenter and consensus scores had a linear-weighted kappa 0.95 ± 0.05.

### 3.3. Tumor Board Management Decisions

**Figure 3:**
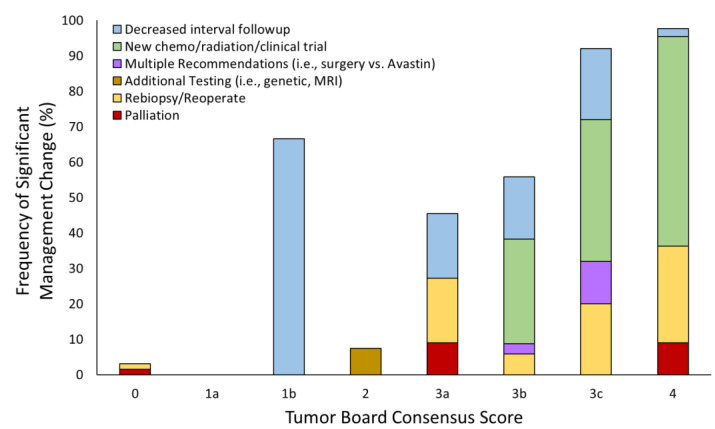
The percent changes in management recommended by tumor board based on the consensus score. The relative distributions of the specific changes recommended are shown as well. For those not shown, no management change was recommended.

The rates of change in management recommendations based on TB consensus scores ranged from 0% for a score of 1a to 97.7% for a score of 4 (Figure 3). When compared with the rate of management changes for a score of 2 (stable imaging), there was a significant difference between rate of management changes for scores 1b (*p* = 0.0485), 3b (*p* = 0.0002), 3c (*p* < 0.0001), and 4 (*p* < 0.0001). Score 0 recommendations included re-biopsy/re-operation (1.5%) and palliative care (1.5%). Score 1a had no changes in management. Score 1b had recommendations for decreased interval follow-up (67%). A score of 2 noted an 8.3% total change in management, all recommending additional testing (namely, additional genetic testing and repeat MRI). The recommendations for score 3a included palliation (7.7%), re-biopsy/re-operate (15.4%), and decreased interval follow-up (15.4%). The changes in management for 3b included new chemotherapy/radiation/clinical trial (29.4%), decreased interval follow-up (17.7%), re-biopsy/re-operate (5.9%), and multiple recommendations (i.e., surgery vs. Avastin) (2.9%). Score 3c recommendations included new chemo/radiation/clinical trial (40.0%), decreased interval follow-up (20.0%), and re-biopsy/re-operation (20.0%), and multiple recommendations (12.0%). A total of 97.7% of patients with a score of 4 had recommended management changes including chemotherapy/radiation/clinical trial (57.8%), re-biopsy/re-operate (26.7%), palliative care (8.9%), and decreased interval follow-up (2.2%).

### 3.4. Clinical Implementation and Outcomes

There were 184 (86.8%) cases that had clinical follow-up within 90 days after the tumor board. Of these, 155 (84.2%) of the recommendations were implemented. Twenty-six (14.1%) of the clinical follow-ups varied from the TB recommendations, most commonly with new chemotherapy initiated in clinic (*n* = 12, 46.2%). The additional breakdown of these implementations is mentioned in Table 3. 

There were 17 individuals who underwent an additional biopsy or re-resection (Table 4). Patients with a consensus score of 0–2 had a lower rate of repeat surgery (2/95, 2.1%) compared with those with a score of 3–4 (12/117, 10.3%, *p* = 0.02). Fourteen (14/17, 82.4%) of the patients undergoing repeat surgery had a significant proportion of tumor (>50%) on the repeat pathology specimen, with the rest representing predominantly radiation necrosis. Only three biopsies had results indicating <50% tumor proportion, namely, one each for score 3b, 3c, and 4. There were no significant differences between groups.

There were 27 confirmed patient deaths within 12 months of the TB, with 23 occurring within 6 months. Mortality at 12 months by tumor consensus score was 0—13.8%, 1a—0%, 1b—0%, 2—4%, 3a—30.8%, 3b—14.7%, 3c—4.0%, and 4—15.6%. Of these, only 3a had a statistically significant difference in mortality compared with those with score 2 (*p* = 0.04).

**Figure 4:**
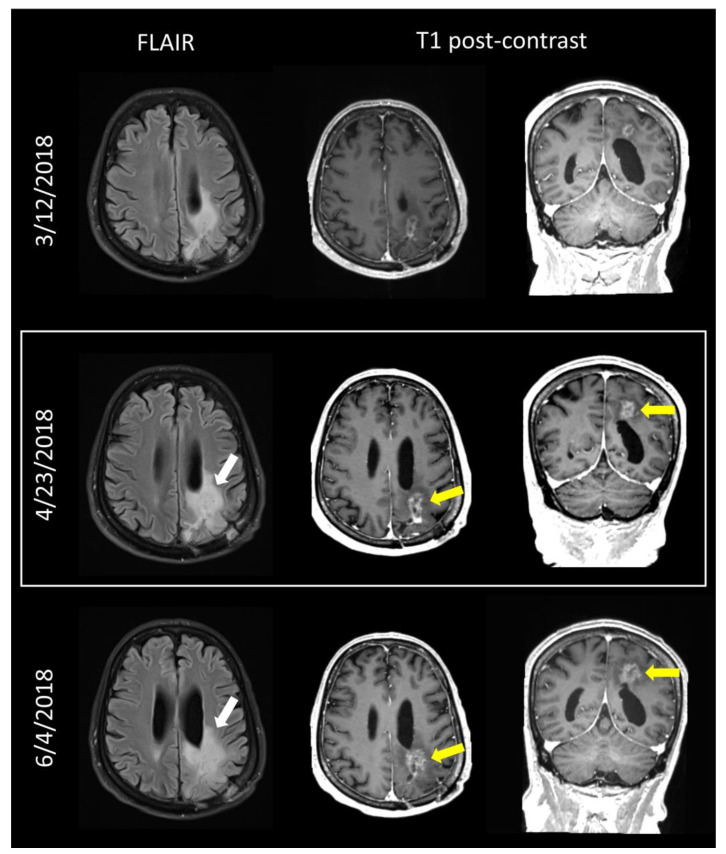
Images from a 71 year-old man with IDH wild-type glioblastoma. Images from 4/23/2018 (middle row, white box) were reviewed at tumor board. Compared with prior study (top row), both FLAIR (white arrow) and enhancing tumor volume were increasing (yellow arrows). While the radiology report called this study a 3b, the tumor board presenter and the tumor board consensus felt the increase was sufficient to score it as disease progression (BT-RADS 4). Subsequent follow-up (bottom row) showed further increase (arrows) and the patient underwent re-irradiation of recurrent disease.

Figure 4 shows sample images from a patient with a discrepancy between the original report and tumor board review. This patient was diagnosed with a glioblastoma, IDH wild type, MGMT unmethylated, in January 2016 and completed primary chemoradiation in March 2016. Tumor board review was completed in April 2018. The initial report was assigned a score of 3b (indeterminate mix of treatment effects and tumor), while both the tumor board presenter and tumor board consensus felt that there was a sufficient increase to assign a score of 4 (tumor progression) because of a greater than 25% in axial tumor area, similar to RANO progression criteria. The patient had continued progression on a follow-up study and underwent re-irradiation in July 2018.

## 4. Discussion

Tumor boards are an important part of cancer care, as they allows clinical decisions to be made in a multidisciplinary environment with input from multiple involved subspecialty experts. Imaging plays a key role in TB evaluation and decision-making, but its impact is challenging to quantify given the qualitative and varied nature of imaging reporting. The use of a structured imaging scoring system, in this case BT-RADS, provides an opportunity to quantify imaging outcomes in a way that has not been previously reported. Assignment of specific categories or scores tied to likely management recommendations allows for prospective quantitation of consistency, accuracy, and relationship to outcomes. Furthermore, clear criteria for how scores are assigned and availability of easy-to-use scoring resources makes structured reporting easy to implement in a multidisciplinary setting [25]. Communication between radiologists and other subspecialists involved in brain tumor care improves [22], especially as other TB participants come to appreciate the common lexicon used. Use of such a system can potentially increase the value of radiologists participating in patient care and decrease the amount of time required for report generation and tumor board preparation. Additionally, structured assessment would potentially be valuable for longitudinal patient registry data collection so that patients with the same disease can be tracked in a more consistent manner.

The most common cases reviewed at the TB were those getting primary evaluation (score 0) to make decisions about initial management and those highly suspected of worsening (score 4), for which a change in therapy was likely needed. There was overall strong agreement between all three interpretation scores, with agreement rates from 79.0 to 90.1% and kappa from 0.86 to 0.95. There was comparatively higher agreement among the presenter and consensus scores as compared with the report and consensus scores. Because only a subset of neuroradiologists participate in the tumor board, these presenters’ interpretations likely converged through feedback among themselves and other TB participants, whereas other reporting radiologists did not routinely receive similar feedback. Additionally, other tumor board participants may be more likely to trust the neuroradiologists they interact with on a regular basis. New clinical information is also often available at the time of presenter review that was not available at the time of original review. Disagreement rates are higher for worsening imaging findings (scores 3–4), which may be more prone to subjective interpretation differences. One advantage of BT-RADS is the classification of worsening findings into unambiguous categories based on perceived etiology in an attempt to minimize disagreement and ambiguity [16,17,21]. 

Higher consensus BT-RADS scores were associated with higher rates of management change recommendations, similar to the literature, in which more benign findings had comparatively lower rates of changes in management than more malignant ones [26]. Lower scores were also associated with less drastic changes in management, such as shorter interval follow-up, as opposed to higher scores, which were more associated with repeat surgery or palliative care. While these trends are expected, without a structured scoring system they are not easily quantified. Consequently, there are no comparable management change data prior to the implementation of BT-RADS at our tumor board. 

Recommendations made by the TB were implemented in the vast majority of cases, in line with the range of 27–91% seen in the literature for other tumor boards [8,9,26]. Variations from the recommendations often account for patient preference and other factors such as patient co-morbidities [10]. Most frequently, management was shifting towards short-term follow-up or new chemotherapy. Higher consensus scores (3–4) were associated with higher rates of repeat surgery or biopsy, and most biopsies performed reflected predominantly tumor. Consensus scores were largely not predictive of patient mortality. This may reflect the heterogeneity of the patient population, including pathologic diagnosis, genetic features, and tumor location, as another study of BT-RADS scores in glioblastoma was predictive of mortality [27]. Higher mortality in 3a patients in the study may reflect selection bias, as patients with worsening outcome were more likely to be presented in the tumor board. Further study of a larger group is needed to understand the correlation of scores and patient mortality, and incorporating advanced techniques such as diffusion weighted imaging and perfusion imaging may improve stratification of 3a patients [28].

There are several limitations to this study. This single-institution analysis may not reflect the broader population, and brain tumor management decisions can be highly institution-specific. Cases selected for the tumor board are typically more complex and may have more confounding factors, which may inflate disagreement rates. Repeat scoring was not blinded, and when the radiology reports did not include a prospectively assigned score it was assigned by the presenting radiologist using information in the report, both of which can introduce bias in agreement rates. It might have been better to blind the reader to the original report, although this may not be practical in a clinical work setting. Further study in a broader group of cases will be needed to fully assess blinded inter-rater agreement of BT-RADS, but this is likely a useful estimate of real-world performance in a tumor board setting. This analysis only includes imaging interpretation, while sometimes tumor board decisions may depend on other factors including tumor genetics (such as IDH, MGMT, and 1p19q status), radiation treatment plans, or patient functional status [5,6,7,29], which were not fully explored within this analysis. Only limited conclusions can be drawn from biopsy and mortality outcomes in this relatively heterogeneous group of cases. 

In conclusion, structured reporting systems can provide unambiguous classifications that allow quantification of the impact of imaging on brain TBs and associated management decisions. There is relatively strong score agreement in the tumor board setting, and higher structured report scores are associated with greater rates of management change. These results suggest the potential value of incorporating this type of classification approach across different institutions, potentially modifying it to fit local practice patterns. Further studies with larger patient sets, multiple institutions, and longer follow-up will help us better understand the true impact of TBs and their effects on rates of mortality among patients with brain tumors. 

## 5. Conclusions

In conclusion, structured reporting systems can provide unambiguous classifications that allow quantification of the impact of imaging on brain TBs and associated management decisions. There is relatively strong score agreement in the tumor board setting, and higher structured report scores are associated with greater rates of management change. Further studies with larger patient sets, multiple institutions, and longer follow-up will help us better understand the true impact of TBs and their effects on rates of mortality among patients with brain tumors. 

## Figures and Tables

**Table 1:** BT-RADS Scoring Classification.

Score	Title	Subscore	Description
0	Not scored		New baseline, incomplete study, or otherwise unable to categorize
1	Imaging improving	1a—Improvement	Improvement in imaging findings suspected to reflect decreasing tumor burden and/or improving treatment effect
		1b—Medication effect	Improvement in imaging findings potentially due to effect from medications such as increasing steroids or initiating Avastin
2	No change		No appreciable change from the prior
3	Imaging worsening	3a—Favor treatment effect	Worsening imaging findings favored to represent treatment effects, including radiation therapy and medications
		3b—Indeterminate	Worsening imaging findings favored to represent an indeterminate mix of treatment effect and tumor worsening
		3c—Favor tumor progression	Worsening imaging findings favored to represent increasing burden of tumor
4	Imaging worsening		Worsening imaging findings highly suspicious for tumor progression

Table shows the scoring criteria for assigning BT-RADS scores used in the study. Criteria have been published previously [21], with additional details available at www.btrads.com.

**Table 2:** Patient and Tumor Characteristics.

Category	Number (%)
Patients	130
Age Range	23–84 years
Age Mean	55 years
Age Median	57 years
Tumors	
Glioblastoma	63 (48.5)
Anaplastic astrocytoma (grade 3)	16 (12.3)
Oligodendroglioma (grade 2)	12 (9.2)
Astrocytoma (grade 2)	11 (8.5)
Anaplastic oligodendroglioma (grade 3)	10 (7.7)
Gliosarcoma	4 (3.1)
Other	
Presumed glioma (no biopsy)	6 (4.6)
Anaplastic pleomorphic xanthoastrocytoma	1 (0.8)
Medulloblastoma	1 (0.8)
Grade 1 glioneural tumor, G1	1 (0.8)
Other diffuse astrocytoma	1 (0.8)
Ependymoma	1 (0.8)
Diffuse midline glioma	1 (0.8)
Atypical liponeurocytoma	1 (0.8)
Grade 1 glioma	1 (0.8)

Data show the characteristics of patients included in the study. Glioblastomas comprised nearly half of the cases, with the remainder a mix of low–intermediate grade gliomas and rarer parenchymal tumors. Meningiomas and metastatic disease were not included in the study.

**Table 3:** Tumor board recommendation implementation.

Category	Number (%)
Total Cases	212
Clinical follow-up within 90 days	184 (86.8)
Clinicians implemented TB recommendations	155 (84.2)
Clinicians did not implement TB recommendations	26 (14.1)
New chemotherapy instead of TB recommendation	12 (5.7)
Decreased interval follow-up instead of TB recommendation	7 (3.3)
Continue follow-up/no change instead of TB recommendation	3 (1.4)
Palliative care instead of TB recommendation	2 (0.9)
Clinical trial instead of TB recommendation	1 (0.5)
Re-operate/re-biopsy instead of TB recommendation	1 (0.5)
Patient changed provider after clinical visit	3 (1.6)
Clinical follow-up >90 days	18 (8.5)
No follow-up present after tumor board	10 (4.7)
No follow-up due to death following tumor board	4 (1.9)

TB—tumor board. Data show the number and percentage of the total patients who had tumor board recommendations after review.

**Table 4:** Distribution of Re-biopsies by Score and Tumor Percentage.

TB Consensus Score	# Biopsied (% Biopsied of All Similar Consensus Scores)	Percent of Biopsies with >50% Tumor Proportion
0	1 (1.5)	100
2	1 (3.7)	100
3a	1 (9.1)	100
3b	1 (2.9)	50
3c	2 (8.0)	67
4	8 (18.2)	89

Rates of re-biopsy/re-operation by BT-RADS consensus score. Rates of biopsy increased with increasing BT-RADS score. The vast majority of patients undergoing repeat surgery had significant recurrent tumor.

## Data Availability

Upon request, the original deidentified data will be provided through request to the corresponding author.
